# Genetic profiling and cardiovascular phenotypic spectrum in a Chinese cohort of Loeys-Dietz syndrome patients

**DOI:** 10.1186/s13023-019-1282-3

**Published:** 2020-01-08

**Authors:** Hang Yang, Yanyun Ma, Mingyao Luo, Guoyan Zhu, Yinhui Zhang, Binbin Li, Chang Shu, Zhou Zhou

**Affiliations:** 1 State Key Laboratory of Cardiovascular Disease, Beijing Key Laboratory for Molecular Diagnostics of Cardiovascular Diseases, Diagnostic Laboratory Service, Fuwai Hospital, National Center for Cardiovascular Diseases, Chinese Academy of Medical Sciences and Peking Union Medical College, Beijing, 100037 China; 2 State Key Laboratory of Cardiovascular Disease, Center of Vascular Surgery, Fuwai Hospital, National Center for Cardiovascular Diseases, Chinese Academy of Medical Sciences and Peking Union Medical College, Beijing, 100037 China

**Keywords:** Loeys-Dietz syndrome, Genetic testing, Phenotypic spectrum

## Abstract

**Background:**

Loeys-Dietz syndrome (LDS) is a rare connective tissue disorder for which 6 genes in the TGF-β pathway have been identified as causative. With the widespread use of genetic testing, the range of known clinical and genetic profiles has broadened, but these features have not been fully elucidated thus far.

**Methods and results:**

Using gene panel sequencing or whole exome sequencing, we identified 54 unique rare variants in LDS genes in 57 patients with thoracic aneurysms/dissections, including 27 pathogenic mutations (P + LP) and 27 variants of unknown significance (VUS^LP^ + VUS). Genotype-phenotype correlation analysis revealed that carriers with P/LP/ VUS^LP^ variants in *TGFBR1*/*TGFBR2*/*SMAD3* genes had significantly more severe cardiovascular features (cardiovascular death/dissection) than carriers with VUSs in these 3 genes at an early age and had less favorable event-free survival. Additionally, carriers with VUS in combination with other risk factors, such as hypertension, might be prone to developing an aortic dissection, as indicated by the fact that 5/8 (62.5%) patients with VUSs in our cohort developed aortic dissections in the presence of hypertension, compared with 25.0% (3/12) in the absence of hypertension (*p* = 0.047).

**Conclusions:**

To date, this was the largest cohort of LDS patients ever reported in China, and the present study expanded the known mutation and phenotypic spectra of LDS, which might help refine our knowledge of LDS.

## Background

Loeys-Dietz syndrome (LDS) is an autosomal dominant connective tissue disorder characterized by cardiovascular deformity (aortic aneurysms and/or dissections, multiple arterial aneurysms and arterial tortuosity) and skeletal problems (arachnodactyly, pectoral deformities, scoliosis and joint laxity) as well as other craniofacial and cutaneous abnormalities, sharing some features with Marfan syndrome (MFS) and differing in other respects [1]. Initially, LDS was generally thought to be more aggressive than MFS, with dissections at younger ages and at smaller arterial diameters, which led to a lower threshold (4.2 cm) for prophylactic surgical intervention by the 2010 American Heart Association (AHA) guideline [2].

Mutations in *TGFBR1* and *TGFBR2* were discovered in 2005 as the first known causative mutations for LDS [1]. Subsequently, other genes in the TGF-β signaling pathway, including *SMAD3* [3], *TGFB2* [4, 5], *SMAD2* [6] and *TGFB3* [7], were also found to be associated with LDS. Subsequently, the definition of LDS expands to all patients who carry a heterozygous pathogenic variant in any of these six genes in combination with the presence of an artery aneurysm/dissection or corresponding systemic manifestations.

The full spectrum of phenotypes and mutations associated with *TGFBR1*- and *TGFBR2*-related LDS has been extensively described and well recognized. However, for the more recently identified LDS genes (*TGFB2*, *TGFB3* and *SMAD2*, *SMAD3*), the phenotypic and genotypic spectra have not yet been fully elucidated and need further expansion. Current studies reveal that patients with *TGFB2*-, *TGFB3*- and *SMAD2*-related LDS tend to have mild cardiovascular features and that their mutations have lower penetrance than those that cause *TGFBR1*- and *TGFBR2*-related LDS [4, 7, 8]. Therefore, further clinical and genetic data on LDS from around the world should be collected and analyzed to define gene-specific vascular treatment guidelines for LDS, rather than treating them all with the same approach. In this study, we identified 54 unique rare variants in LDS genes in aortic aneurysm/dissection patients and summarized the clinical data of these patients, especially their vascular phenotypic data, which could help further refine our knowledge of LDS.

## Materials and methods

### Patients

More than 900 patients with aortic disease and/or diagnosed or suspected MFS had been referred from the Aortic Surgery Department to the Center for Molecular Diagnosis at Fuwai Hospital and had undergone panel testing involving 15 genes (*ACTA2, COL3A1, FBN1, FBN2, MYH11, MYLK, NOTCH1, PRKG1, SKI, SLC2A10, SMAD3, SMAD4, TGFB2, TGFBR1, TGFBR2*) since Feb 2014 [9]. Furthermore, more than 200 aortopathy patients were performed whole exome sequencing. From these patients, we included a total of 57 patients in this study, in whom a rare variant in any of the six genes *TGFBR1, TGFBR2*, *SMAD3*, *TGFB2, SMAD2* and *TGFB3* was detected with no other suspected causative mutations.

### Variant classification

Variants were analyzed for pathogenicity in line with recommendations from the American College of Medical Genetics (ACMG) and classified into one of 5 categories: benign, likely benign, unknown significance, likely pathogenic or pathogenic [10], with the detailed evidences listed behind. Besides, we additionally defined a subclassification, VUS^LP^, for internal use (See “Results” section for details).

### Statistical analysis

Statistical analyses were performed using SPSS software. Survival curves were estimated using the Kaplan–Meier method and tested by Log Rank tests. Comparisons between continuous variables were made by Student’s t-test. A one-tailed Chi-Square test was used to test if the presence of hypertension facilitated aortic dissections in patients with VUSs in LDS genes. *P* value below 0.05 was considered as statistically significant.

## Results

**Fig. 1:**
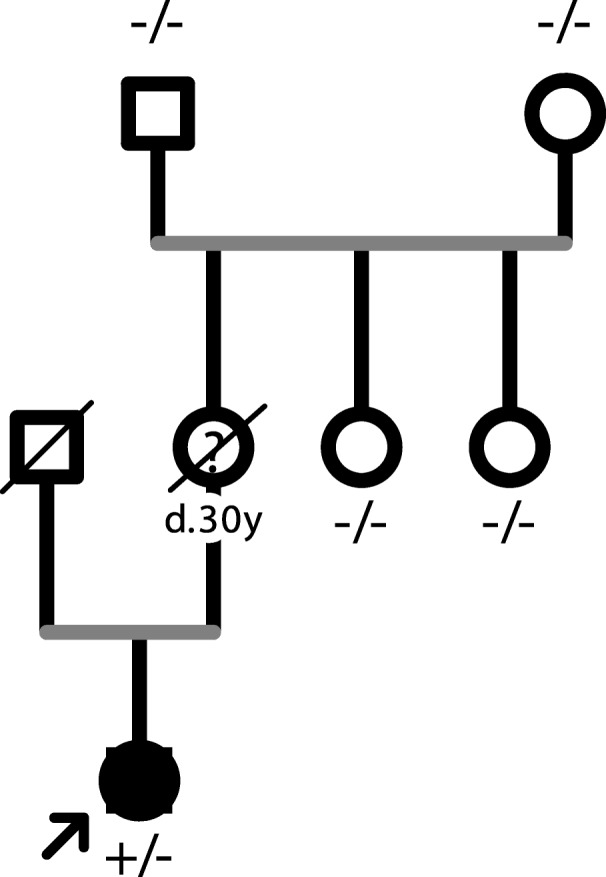
The pedigree of patient AD1181. Black indicated affected while white indicated unaffected.? represented that the person was suspected to have a sudden death due to an aortopathy

Among all of the aortopathy patients, a total of 54 unique rare variants in LDS genes were identified in 57 separate patients. Of these variants, 27 were pathogenic or likely pathogenic (summarized in Table 1), mostly in *TGFBR1* and *TGFBR2* genes (10 in *TGFBR1*, 10 in *TGFBR2*), and 24 variants remained unknown significance (summarized in Table 2). Specifically, there were 3 variants which should be classified as VUS on account of lack of evidence according to ACMG criteria. However, they were highly suspected as causative in the light of clinical information and family history. Therefore, we additionally defined a subclassification for these variants, VUS^LP^, for internal use (Table 1). The variants which could meet the tier “Likely Pathogenic” in ACMG criterion with one more supporting evidence, or those which could be assumed to be de novo according to the family history, were classified as VUS^LP^. For instance, patient AD1181’s mother suffered a sudden cardiac death at 30, therefore we could not collect her sample to perform the gene testing. However, there was a high probability that she carried the same mutation with her daughter AD1181, which was a de novo mutation because her parents and two sisters were all healthy and did not carry the mutation (Fig. 1).

**Table 1:** Definite pathogenic and highly suspected variants in LDS genes detected in our cohort

Patient ID	Gene	Transcript	Nucleotide change	Amino acid change	MAF in ExAC	MAF in gnomAD	Domain	Source	Pathogenicity	Evidence	Note
AD1413	*TGFBR1*	NM_004612	c.614 T > C	p.Ile205Thr	.	.	Pkinase_Tyr	Maternal	LP	PM2, PP3, PS2^$^	
AD623–1	*TGFBR1*	NM_004612	c.644G > C	p.Arg215Pro	.	.		De novo	LP	PS2, PM2, PP3	
AD808	*TGFBR1*	NM_004612	c.664G > A	p.Gly222Arg	.	0.0000289	Pkinase_Tyr		LP	PM2, PP1_Strong, PP3	
AD264	*TGFBR1*	NM_004612	c.683_685del	p.228del	.	.		De novo	LP	PS2, PM2, PS4_Supporting, PM4	^a^
AD692–1	*TGFBR1*	NM_004612	c.702_704del	p.235del	.	.		De novo	LP	PS2, PM2, PM4	
AD453	*TGFBR1*	NM_004612	c.722C > T	p.Ser241Leu	.	.		NA	LP	PM2, PS4_Supporting, PS2	
AD371	*TGFBR1*	NM_004612	c.934G > A	p.Gly312Ser	0.00000942	0.00000398		NA	LP	PP3, PM2, PS4_Supporting, PP1_Strong	^a^
AD641–1	*TGFBR1*	NM_004612	c.997G > A	p.Asp333Asn	.	.		De novo	LP	PS2, PM2, PP3	
AD78	*TGFBR1*	NM_004612	c.1459C > T	p.Arg487Trp	.	.		NA	P	PS4, PM2, PM5, PP1_Strong, PP3	^a^
AD703–1	*TGFBR1*	NM_004612	c.1459C > T	p.Arg487Trp	.	.		Maternal	P	PS4, PM2, PM5, PP1_Strong, PP3	
AD1346	*TGFBR1*	NM_004612	c.1459C > T	p.Arg487Trp	.	.		NA	P	PS4, PM2, PM5, PP1_Strong, PP3	
AD1362	*TGFBR1*	NM_004612	c.1460G > A	p.Arg487Gln	.	.		Paternal	P	PS2, PS3_Supporting, PS4_Moderate, PM2, PP3	
AD1804	*TGFBR2*	NM_003242	c.95-2A > G		0.0000293	0.0006		Paternal	LP	PVS1, PM2	
AD257	*TGFBR2*	NM_003242	c.1067G > C	p.Arg356Pro	.	.	Pkinase_Tyr	Paternal (Mosaic)	P	PS2_Very Strong, PS4_Moderate, PM2, PP3	^ab^
AD22	*TGFBR2*	NM_003242	c.1139 T > G	p.Leu380Arg	.	.		De novo	LP	PS2, PM2, PP3	^a^
AD888	*TGFBR2*	NM_003242	c.1275G > C	p.Met425Ile	.	.	Pkinase_Tyr	De novo	LP	PS2, PM2, PP3	
AD1181	*TGFBR2*	NM_003242	c.1363 T > C	p.Trp455Arg	.	.	Pkinase_Tyr		VUS^LP^	PM2, PP3	Assumed de novo in AD1181’s mother
AD536	*TGFBR2*	NM_003242	c.1449dupT	p.Cys483fs	.	.	Pkinase_Tyr	NA	LP	PVS1, PM2	
AD617–1	*TGFBR2*	NM_003242	1517delA	p.Asn506fs	.	.	Pkinase_Tyr	NA	P	PVS1, PM2, PP1	
AD1784	*TGFBR2*	NM_003242	c.1525-1G > C		.	.		NA	LP	PVS1, PM2	
AD153	*TGFBR2*	NM_003242	c.1538 T > C	p.Val513Ala	.	.	Pkinase_Tyr	De novo	LP	PS2, PM2	^a^
AD682–1	*TGFBR2*	NM_003242	c.1582C > T	p.Arg528Cys	.	.		De novo	P	PS2, PP3, PM2, PS4_Moderate, PS3_Supporting, PM5	
AD497	*TGFBR2*	NM_003242	c.1609C > T	p.Arg537Cys	.	.		NA	P	PS2, PS3_Moderate, PS4_Moderate, PM2,PP3, PP1_Strong	^a^
AD1550	*SMAD3*	NM_005902	c.233_234insGG	p.Ser78fs	.	.		NA	LP	PVS1, PM2	
AD1736	*SMAD3*	NM_005902	c.365_366insGAATCCCTACCAC	p.Val122fs	.	.		Paternal	LP	PVS1, PM2	
AD1061	*SMAD3*	NM_005902	c.1041delG	p.Glu347fs	.	.		NA	LP	PVS1, PM2	
AD792	*SMAD3*	NM_005902	c.1118G > A	p.Arg373His	.	.			VUS^LP^	PM2, PP3, PS3_Supporting, PS4_Supporting	
AD1297	*SMAD3*	NM_005902	c.1247C > T	p.Ser416Phe	.	.		De novo	LP	PS2, PM2, PP3	
AD535	*SMAD2*	NM_005901	c.593dupA	p.His198fs	.	.		De novo	LP	PS2, PM2	
AD802	*TGFB2*	NM_003238	c.905G > A	p.Arg302His	.	.	TGF_beta	Paternal	VUS^LP^	PM2, PM5, PP3	
AD1065	*TGFB3*	NM_003239	c.605_623del	p.Phe202fs	.	.		Maternal	LP	PVS1, PM2	
AD631–1	*TGFB3*	NM_003239	c.646 + 2 T > G		.	.		Paternal	LP	PVS1, PM2	

Note: *NA* not available; MAF in ExAC was the maximal allele frequency from the public version (20160423), and MAF in gnomAD was the maximal allele frequency from gnomAD v2.1.1; P, pathogenic; LP, likely pathogenic; VUS, variant of uncertain significance; ^a^, reported in our previous article [11]; ^b^This variant was previously classified as VUS, and then upgraded into pathogenic after the father was confirmed to carry a mosaic mutation in the same site; $ This variant was confirmed to be de novo in patient AD1413’s mother

**Table 2:** Variants of unknown significance in LDS genes detected in our cohort

Patient ID	Gene	Transcript	Nucleotide change	Amino acid change	MAF in ExAC	MAF in gnomAD	Domain	Source	Pathogenicity	Evidence	Note
AD1039	*TGFBR1*	NM_004612	c.341C > G	p.Thr114Ser	.	.			VUS	PM2, BP4	
AD1248	*TGFBR1*	NM_004612	c.439A > G	p.Ile147Val	0.0000221	0.000098	Pkinase_Tyr		VUS	BP4	
AD589	*TGFBR1*	NM_004612	c.605C > T	p.Ala202Val	.	.			VUS	PM2, PP3	
AD823	*TGFBR1*	NM_004612	c.767A > G	p.His256Arg	.	.			VUS	PM2, PP3	
AD1802	*TGFBR1*	NM_004612	c.782G > C	p.Gly261Ala	.	.			VUS	PM2, PP3	
AD183	*TGFBR1*	NM_004612	c.929C > T	p.Ala310Val	0.0000221	0.0006			VUS	PP3	
AD436	*TGFBR1*	NM_004612	c.935G > T	p.Gly312Val	.	.			VUS	PM2, PP3	
AD1158	*TGFBR1*	NM_004612	c.1054 T > G	p.Leu352Val	.	.			VUS	PM2, PP3	
AD1753	*TGFBR2*	NM_003242	c.81C > A	p.His27Gln	.	.		NA	VUS	PM2, BP4	
AD1432	*TGFBR2*	NM_003242	c.467G > T	p.Ser156Ile	.	.			VUS	PM2, BP4	
AD1348	*TGFBR2*	NM_003242	c.578G > A	p.Arg193Gln	0.000011	0.0000544	TGF_beta	Paternal	VUS	PM2	
AD1156	*TGFBR2*	NM_003242	c.617C > T	p.Thr206Met	0.0000377	0.0006			VUS	BP4	
AD259	*TGFBR2*	NM_003242	c.830A > G	p.Lys277Arg	.	.	Pkinase_Tyr		VUS	PM2, PP3	^a^
AD667	*TGFBR2*	NM_003242	c.1188 T > G	p.Cys396Trp	.	.	Pkinase_Tyr		VUS	PM2, PP3	
AD1162	*TGFBR2*	NM_003242	c.1254G > T	p.Gln418His	.	.	Pkinase_Tyr	Maternal	VUS	PM2, PP3	
AD324	*SMAD3*	NM_005902	c.5C > T	p.Ser2Leu	.	.		Paternal	VUS	PM2	^ab^
AD1250	*SMAD3*	NM_001145103	c.53G > A	p.Arg18Gln	.	.			VUS	NA	
AD76	*SMAD3*	NM_005902	c.140_148del	p.47_50del	.	.			VUS	PM2, PM4, BS2	^a^
AD997	*SMAD3*	NM_005902	c.364G > A	p.Val122Met	.	.			VUS	PM2, PP3	
AD850	*SMAD3*	NM_005902	c.773A > T	p.Asp258Val	.	.			VUS	PM2, PP3	
AD1288	*SMAD3*	NM_005902	c.1027 T > C	p.Phe343Leu	.	.			VUS	PM2, PP3	
AD148	*SMAD3*	NM_005902	c.1027 T > C	p.Phe343Leu	.	.			VUS	PM2, PP3	
AD1759	*TGFB2*	NM_003238	c.893G > A	p.Arg298Gln	0.0000221	0.0002		NA	VUS	NA	
AD1599	*TGFB2*	NM_003238	c.1239C > G	p.Cys413Trp	.	.			VUS	PM2, PP3	
AD985	*TGFB3*	NM_003239	c.352 + 5G > A		.	.			VUS	PM2, PP3	

Note: *NA* not available; MAF in ExAC was the maximal allele frequency from the public version (20160423), and MAF in gnomAD was the maximal allele frequency from gnomAD v2.1.1; P, pathogenic; LP, likely pathogenic; VUS, variant of uncertain significance; ^a^, reported in our previous article [11]; ^b^This variant was previously misclassified as likely pathogenic [11], and now corrected into VUS

**Fig. 2:**
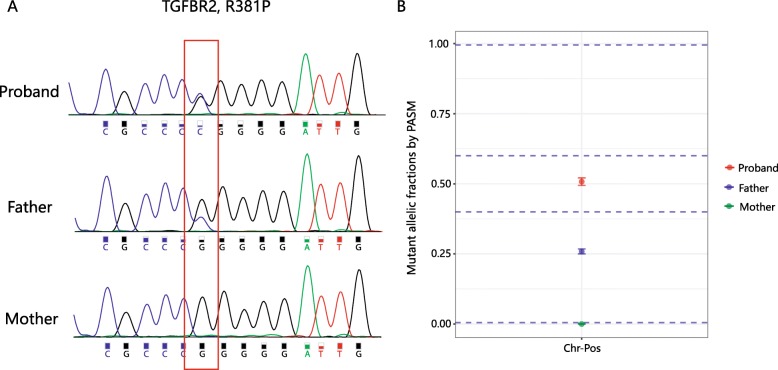
Mosaicism in patient AD257’s family. **a**, Sanger sequencing indicated an unequal peak height at the mutation site in patient AD257’s father; **b**, Deep sequencing at the specific location revealed a mosaic mutation in patient AD257’s father

The pathogenicity of the variant *TGFBR2* c.1067G > C (p. Arg356Pro) initially confused us. This variant was identified in patient AD257 with characteristic signs of LDS, such as descending pseudoaneurysm, bilateral carotid tortuosity, bifid uvula and hypertelorism. This variant had been reported in five individuals with clinical features of Loeys-Dietz syndrome and was found to occur de novo in three of these individuals [12–15]. Furthermore, it was absent from large population studies, and computational prediction tools and conservation analysis suggested that it might impact the protein. All evidence supported that it was a pathogenic mutation. However, we unexpectedly found that the patient’s healthy father also carried the same mutation. Upon a detailed examination of the father’s cardiac structure and arterial tree, there were no apparent abnormalities except for a slight decrease in left ventricular diastolic function. Considering that LDS was a dominant disorder with full penetrance expected at an early age and that the variant was also observed in the patient’s healthy father, this variant was finally downgraded into VUS [11] with conflicting evidence (BS2). When we reanalyzed this case after half a year, we noted that the unequal peak heights suggested probable mosaicism. To test this, we performed deep sequencing (5000×) at this location, and the results showed that the father indeed had a mosaic mutation (Fig. 2), which convincingly explained his lack of LDS symptoms.

Patient AD535 had positive wrist sign, pectus excavatum, and moderate myopia. He was found to have an aortic root dilation with a diameter of 45 mm and a bicuspid aortic valve (BAV) upon physical examination. A *SMAD2* frameshift mutation in the linker region was identified and shown to be de novo; therefore, it was classified as a likely pathogenic mutation. According to the genetic results, the patient was diagnosed with Loeys-Dietz syndrome, and an examination of his whole arterial tree was recommended to check for other arterial aneurysms. To our knowledge, this was the second report of a truncating mutation in the *SMAD2* gene and further confirmed haploinsufficiency as its pathogenic mechanism.

**Fig. 3:**
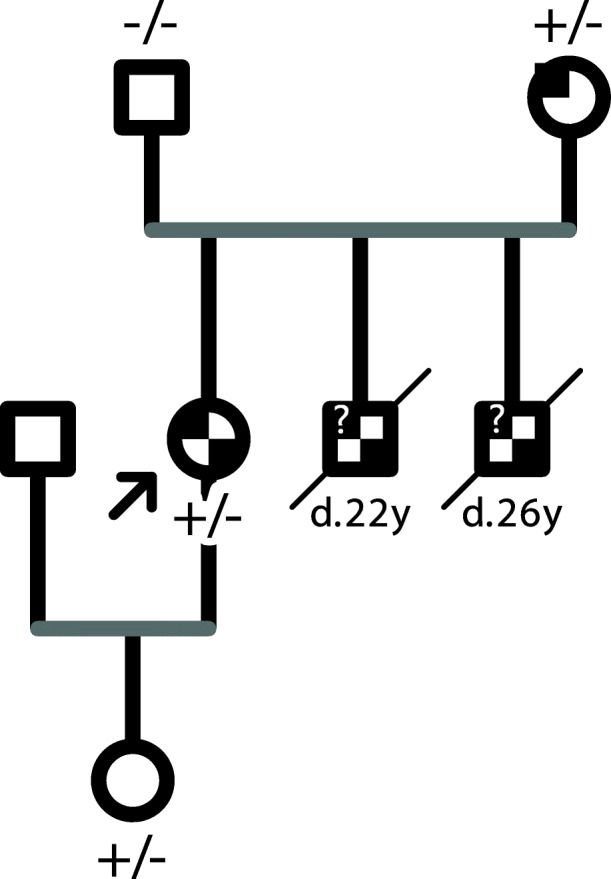
The pedigree of patient AD1162. Black on the top left corner indicated a vascular event, and black on the bottom right corner indicated an ocular event (lens subluxation or retinal detachment).? represented that the person was suspected to have a sudden death due to an aortopathy

Patient AD1162 had an aortic dissection, and also dissections in carotid artery and abdominal aorta. After a detailed investigation, we learnt that the patient had a family history of sudden deaths and retinal detachments (Fig. 3). She had lens subluxation herself. Except that her youngest brother had pectus carinatum and scoliosis, other family members had no obvious skeletal deformities. A 15-gene panel [9] testing revealed that she carried a *TGFBR2* mutation (c.1254G > T, p.Gln418His), which was inherited from her mother, who suffered an aortic dissection at age 42. Therefore, she was highly suspected to be LDS. Strangely, it was commonly thought that one of the most distinguishing characteristics between LDS and MFS was that the former rarely included ocular abnormalities such as ectopia lentis or retinal detachments [16]. To exclude a *FBN1* large deletion/duplication, multiplex ligation-dependent probe amplification (MLPA) assay was also performed, which was negative (Additional file 1: Figure S1). It suggested that there might be a wider spectrum of LDS than we previously realized.

**Fig. 4:**
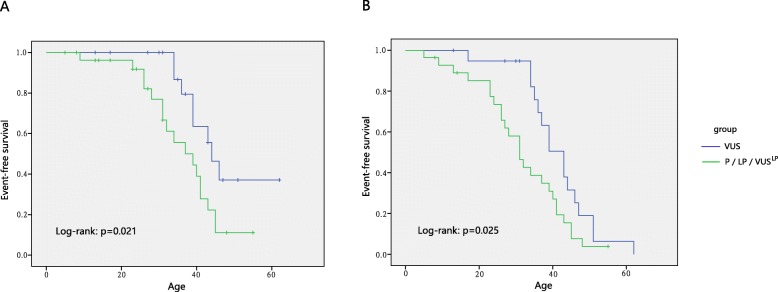
Kaplan–Meier analysis of event-free survival. Event-free survival was compared in probands with P/LP/VUS^LP^ variants (green curve) versus those with VUS (blue curve). **a**, Events were defined as aortic dissections and related deaths; **b**, Events were defined as aortic dissections and related death or aortic surgeries

To analyze the genotype-phenotype correlation, we divided the patients into two groups according to the variant pathogenicity. Considering *TGFBR1/TGFBR2/SMAD3*-related LDS often led to a penetrant and severe form of the disease and accounted for the vast majority in our cohort, only patients with these 3 genes were included to perform the analysis. The key cardiovascular information was listed in Table 3. When patients with P/LP/VUS^LP^ variants were set into one group and the others with VUS were set into another, event-free survival was compared and the results showed that patients with a P/LP/VUS^LP^ variant had a significant lower event-free survival rate than those with VUS (*p* = 0.021 when events defined as aortic dissections or related death; *p* = 0.025 when events defined as dissections and aortic surgeries and related death) (Fig. 4), indicating that the presence of a pathogenic variant has possible predictive value for disease severity. In addition, the presence of (suspected) pathogenic variants was associated with earlier aortic dissection or surgery than the presence of VUS (29.9 y vs 38.0 y, *p* = 0.035). Besides, in 20 individuals with VUSs, 8 patients had hypertension while 12 patients were with normal blood pressure, with a dissection rate of 62.5% (5/8) and 25.0% (3/12), separately (p = 0.047) (Additional file 1: Table S1).

**Table 3:** Main cardiovascular phenotypic information in two subgroups of LDS

	*TGFBR1/TGFBR2/SMAD3*			*TGFB2/TGFB3/SMAD2*		
	LP/P	VUS^LP^	VUS	LP/P	VUS^LP^	VUS
Numbers	26	2	20	3	1	3
Age, years	29.5 ± 13.3	34.0 ± 4.2	38.0 ± 11.5	20.3 ± 10.4	20	43.0 ± 8.5
Normal or mild dilation	3	0	4	3	0	0
Surgery due to an aortic aneurysm/valve disease	8	0	8	0	1	0
Aortic dissection and related death	15	2	8	0	0	3

## Discussion

### Genetic profiling

*TGFBR1* and *TGFBR2* are the first genes identified to be associated with LDS. To date, over 200 variants in these genes have been reported in the human gene mutation database (HGMD), most of which (86%) are missense mutations in evolutionarily conserved residues within the serine-threonine kinase (STK) domains. Missense and truncating variants in the STK domain of the *TGFBR1* gene induce LDS [1] and multiple self-healing squamous epithelioma (MSSE) [17], presumably through the dominant negative (DN) and haploinsufficiency (HI) effects, respectively. For *TGFBR2*, there seems to be no difference in *TGFBR2*-related phenotypic features between individuals with missense mutations and those with truncating mutations [18]. Here, we reported two frameshift mutations and one canonical splicing mutation in the *TGFBR2* gene in three separate individuals who had severe vascular events. Patient AD536 suffered a total thoracic and abdominal replacement at his age of 53 and AD1784 suffered an ascending and full aortic arch replacement at 34, while patient AD617 had a positive family history of sudden cardiac death at an early age.

A total of 78 and 8 variants in *SMAD3* and *SMAD2*, respectively, have been reported in LDS patients [8], the majority (63 and 87.5%, respectively) of which are located in the MH2 domain, a highly conserved region responsible for the oligomerization of SMAD2 or SMAD3 with SMAD4 and subsequent Smad-dependent activation of downstream transcription. Other than missense variants, previous reports reveal only one *SMAD2* nonsense mutation [19], which is located in the linker region. In this study, we identified the second case of a *SMAD2* truncating mutation (c.593dupA), which was also in the linker region. It is very similar to *SMAD3* that all pathogenic mutations in linker regions are truncating mutations [8]. For *TGFB2* and *TGFB3*, 44 and 34 mutations have been reported, respectively, mainly distributed in the TGF-β2 cytokine domain, RKKR-motif and latency-associated peptide (LAP). Here, we reported 3 novel *TGFB3* mutations and 3 novel *TGFB2* mutations.

### Cardiovascular phenotypic spectrum

Significant clinical heterogeneity was observed in LDS patients. When first reported as a distinct disease, LDS was described as having more aggressive aortic events than MFS, with a mean age of 26 years at death [1]. A reduced threshold of 42 mm had been proposed for earlier interventions in LDS patients [2]; however, it remained controversial [20, 21]. Current studies revealed that some patients with *TGFBR1*, *TGFBR2* or *SMAD3* mutations tended to have an early dissection at a young age or at a relatively small diameter, whereas *TGFB2*, *TGFB3* and *SMAD2* carriers often suffered a less severe aortic event [4, 7, 8, 22]. Our data were consistent with previous studies in that all seven individuals with mutations in *TGFB2*, *TGFB3* or *SMAD2* had relatively mild aortic events, except that patient AD985, who had the risk factors of extreme hypertension and an intronic mutation predicted to affect normal splicing, suffered an aortic dissection at 32 years of age.

Considering these two clinical forms in LDS, one more severe and penetrant than another, we only involved the severe form when analyzing the genotype-phenotype correlation, so as to avoid the interpretation bias. The results revealed that carriers with P/LP/VUS^LP^ variants have significantly more severe cardiovascular features (aortic dissection and related death) than carriers with VUS, at an early age and less favorable event-free survival. Notably, according to current evidence, many variants (27, 50%) remain VUS and VUS^LP^. On the one hand, some of these variants may be upgraded to likely pathogenic mutations as further supporting evidence accumulates; on the other hand, these variants may predispose patients to disease in a low-risk or low-penetrance manner and lead to aortic dissection when combined with other risk factors, such as hypertension. This possibility is well supported by our data showing that, when hypertension was present, 5/8 (62.5%) patients with VUS in LDS genes developed aortic dissections, far more than 25.0% (3/12) when hypertension was absent. Based on our current results, carriers with VUSs in LDS genes should receive active control of blood pressure.

## Conclusions

In summary, this was the largest cohort of LDS patients ever reported in China, and we expanded the known mutation and phenotypic spectra of LDS. Genetic results not only facilitate an early and accurate diagnosis but also have possible predictive value, which needs to be further investigated because it may influence clinical care.

## Supplementary information

**Additional file 1. **Method S1. Multiplex ligation-dependent probe amplification (MLPA). **Figure S1.** MLPA assay indicated that there was no *FBN1* deletion/duplication in AD1162. **Table S1.** Main cardiovascular phenotypic information in patients with VUSs in *TGFBR1/TGFBR2/SMAD3* genes.

## Data Availability

The data sets used and analysed during the current study are available from the corresponding author on reasonable request.

## Abbreviations

ACMG: American College of Medical Genetics
AHA: American Heart Association
DN: Dominant negative
ESP: Exome Sequencing Project
HGMD: Human gene mutation database
LAP: Latency-associated peptide
LDS: Loeys-Dietz syndrome
MFS: Marfan syndrome
MLPA: Multiplex ligation-dependent probe amplification
MSSE: Multiple self-healing squamous epithelioma
STK: Serine-threonine kinase
WES: Whole exome sequencing
