# Supplementary material for: Genetic profiling and cardiovascular phenotypic spectrum in a Chinese cohort of Loeys-Dietz syndrome patients
Source: Orphanet J Rare Dis. 2020 Jan 8;15:6. doi: 10.1186/s13023-019-1282-3 (PMC6950884; doi:10.1186/s13023-019-1282-3)
Supplement: Supplementary file 1 — Additional file 1. Method S1. Multiplex ligation-dependent probe amplification (MLPA). Figure S1. MLPA assay indicated that there was no FBN1 deletion/duplication in AD1162. Table S1. Main cardiovascular phenotypic information in patients with VUSs in TGFBR1/TGFBR2/SMAD3 genes. [file 13023_2019_1282_MOESM1_ESM.docx]

**Supplementary Method**

**Multiplex ligation-dependent probe amplification (MLPA)**

MLPA assays were performed to detect *FBN1* and *TGFBR2* large deletions or duplications using the commercially available SALSA MLPA kits P065 and P066 (MRC-Holland, Amsterdam, The Netherlands), which contained probes for all exons of *FBN1* and *TGFBR2*. According to the manufacturer’s instructions, a total of 100-200 ng of genomic DNA of each patient was used for hybridization, and amplification products from each MLPA assay were separated by capillary electrophoresis on an ABI 3500XL Dx Genetic Analyzer (Life Technologies, USA). The results were analyzed using Coffalyser software. Deletions and duplications with deviations more than 30% were suspected as significant alterations.


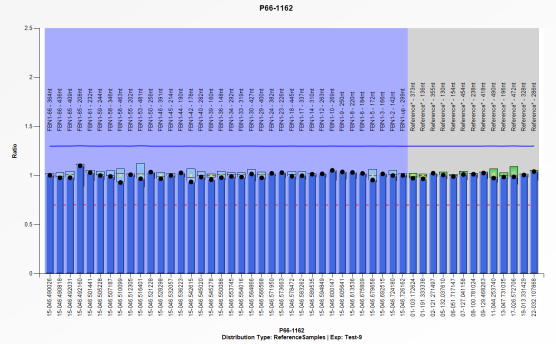
Supplementary Figure 1. MLPA assay indicated that there was no *FBN1* deletion/duplication in AD1162.


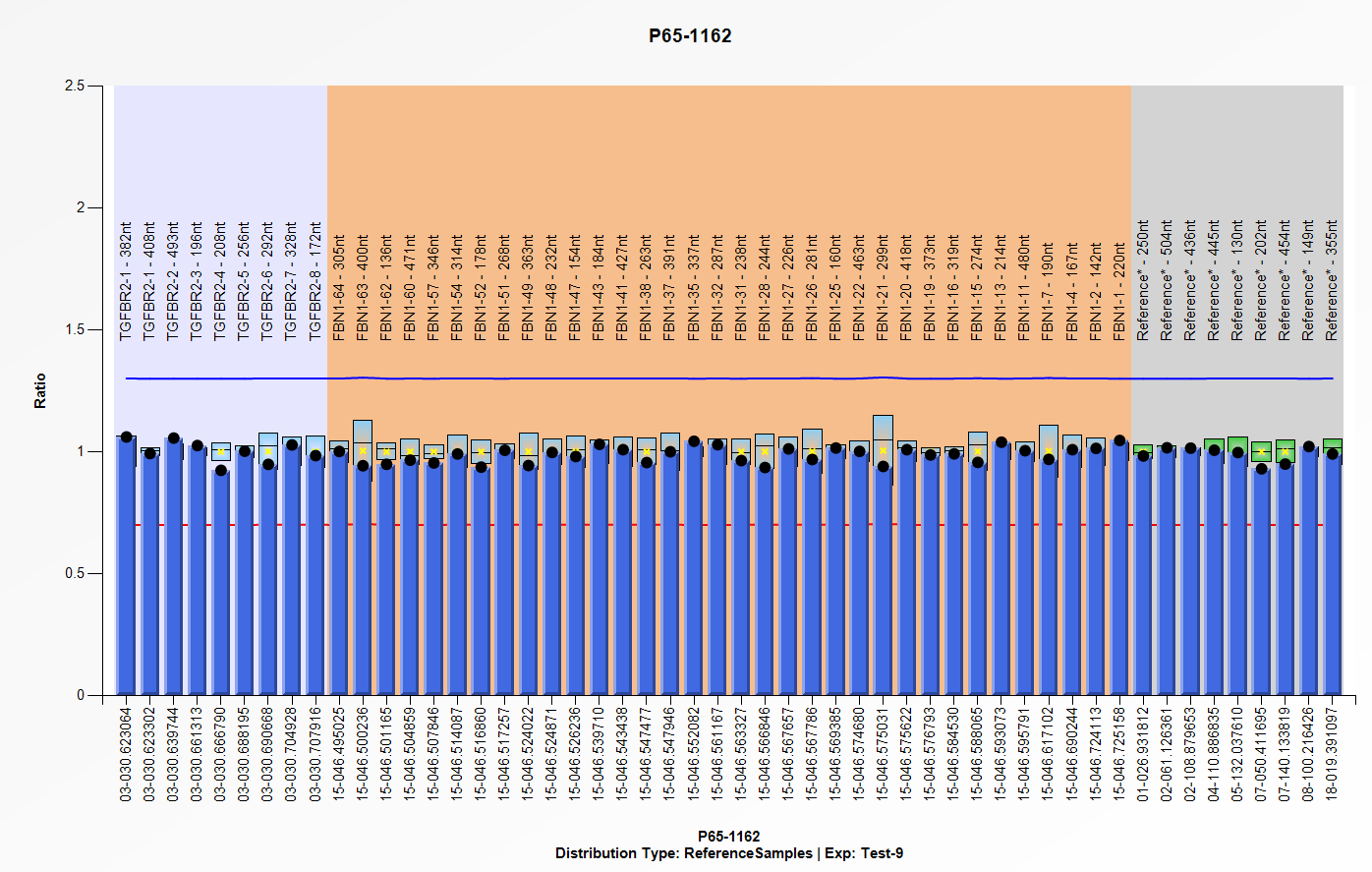


Supplementary Table 1. Main cardiovascular phenotypic information in patients with VUSs in  *TGFBR1/TGFBR2/SMAD3* genes

|  | Patients with VUSs in *TGFBR1/TGFBR2/SMAD3* genes | |
| --- | --- | --- |
|  | With hypertension | Without hypertension |
| Numbers | 8 | 12 |
| Age, years | 42.0 ± 9.2 | 35.3 ± 12.4 |
| Normal or mild dilation | 0 | 4 |
| Surgery due to an aortic aneurysm /valve disease | 3 | 5 |
| Aortic dissection and related death | 5 | 3 |
